# Different effectiveness of anticancer immunotherapy in men and women relies on sex-dimorphism of the immune system

**DOI:** 10.18632/oncotarget.25795

**Published:** 2018-07-27

**Authors:** Fabio Conforti, Laura Pala, Aron Goldhirsch

**Affiliations:** Division of Medical Oncology for Melanoma & Sarcoma, European Institute of Oncology, Milan, Italy

**Keywords:** immune system, sex-dimorphism, sex hormones, immunotherapy, Immune checkpoint inhibitos

In the last ten years Immune checkpoint inhibitors (ICIs) completely changed the therapeutical algorithm for many types of cancer.

Recently, we showed a significant heterogeneity of the efficacy of anti-PD-1 and anti-CTLA-4 drugs in male and female patients with several advanced tumor types [1].

We highlighted the relevance of sex-dimorphism in the effectiveness of ICIs, demonstrating that male and female cancer patients respond in a different way to immunotherapies, regardless of the tumor histological type, the type of treatment and the setting of therapy [1].

The different effectiveness of immunotherapy as anticancer treatment in men and women probably relies on relevant Sex-based differences in immune system function and responses.

On average, adult females mount stronger innate and adaptive immune responses than males [2]. This results in a faster clearance of pathogens, explaining lower severity and prevalence of many infections, and greater response to vaccination [2, 3].

Furthermore, about 80% of systemic autoimmune disease occurs in females [4].

Sex differences in innate immunity were demonstrated in phagocytic efficiency of neutrophils and macrophages, that is greater in females than males, as well as in Antigen-presenting cells (APCs), that are more efficient at presenting peptides in females compared to males [2].

Dissimilarities in adaptive immunity were also described, particularly regarding the relative amount and activity of lymphocyte subsets:

- females have higher CD4+ T cell counts and higher CD4/CD8 ratios than age-matched males; whereas males have higher CD8+ T cell frequencies [2, 5].
- healthy adult males have higher number of T regulatory cells compared with females [2, 5].
- females tend to show greater antibody responses than males, higher basal immunoglobulin levels and higher B cell numbers, regardless of age [2, 5, 6].

Taken together, these data indicate that on average, males have a relative attenuation of immune responses compared with females. These sex-based differences probably reflect complex interactions among genes, hormones and environment.

The X chromosome contains a large number of immune-related genes [2, 5, 7].

These genes code for proteins involved in the regulation of the innate immunity, like pattern recognition receptors (e.g. *TLR7* and *TLR8* ), as well as in the regulation of adaptive immunity, including cytokine receptors (e.g., *IL2RG* and *IL13RA2*) and key transcriptional factors (e.g. *FOXP3*) [2, 7].

Immune-related genes encoded on the X chromosome may escape X inactivation resulting in higher expression levels in females than males [7].

Sex hormones constitute another major determinant of sex differences in immunity.

They modulate the development and function of multiple immune cell populations, shaping innate and adoptive immune responses [2, 8]. Indeed, sexual dimorphism in immune responsiveness is most accentuated post-puberty [2, 8]. Hormonal fluctuations accompanying the menstrual cycle, pregnancy and menopause have a deep impact on susceptibility to infectious disease and autoimmunity [2, 8].

Sex hormones exert potent effects on the regulation of a broad number of immune-related genes in multiple immune cell subsets [8].

Putative androgen response elements (AREs) and estrogen response elements (EREs) are present in the promoters of several innate and adaptive immunity genes, suggesting that sex steroids may directly regulate their expression [8].

Recently, a role for sex-hormones modulation of PD-1/PDL1 pathway has emerged, albeit the published literature is limited to animal studies [8, 9].

Preclinical studies suggest that sex steroids regulate the expression and function of PD-1, and that the hormone-mediated effects on PD-1 pathway is important in mediating autoimmunity [9]. The expression of the PD-1 ligand, PD-L1, has also been shown to be modulated in an estrogen-dependent and sex-dependent manner [9].

Furthermore, recent works have highlighted a tight relation between the commensal microbiome and host sex hormones, showing that the onset of puberty and concurrent hormonal changes result in sex specific microbiome profile [10]. These findings indicate that the hormonal status of the host can modify microbiome composition. Since host microbioma, especially gut microorganisms, strongly contributes to the modulation of immune system, this is a further mechanism by which sex hormones influence the immune sexual dimorphism [10].

Our data demonstrated that such differences in immune systems influence also the modality through which women and men with cancer respond to immunotherapies [1].

Future research should better investigate differences in the molecular mechanisms which govern anticancer immune responses in men and women, in order to optimize new treatments for both.

In our opinion, this will ultimately lead to test different immunotherapeutic strategies in man and woman, tailored on sex-specific features of anticancer immune responses.
